# Hydraulic conductivity and low-density lipoprotein transport of the venous graft wall in an arterial bypass

**DOI:** 10.1186/s12938-019-0669-7

**Published:** 2019-04-25

**Authors:** Zhenze Wang, Ming Liu, Xiao Liu, Anqiang Sun, Yubo Fan, Xiaoyan Deng

**Affiliations:** 1 Beijing Key Laboratory of Rehabilitation Technical Aids for Old-Age Disability, Key Laboratory of Technical Aids Analysis and Identification Key Laboratory of the Ministry of Civil Affairs, National Research Centre for Rehabilitation Technical Aids, Beijing, 100176 China; 2 Key Laboratory for Biomechanics and Mechanobiology of Ministry of Education, School of Biological Science and Medical Engineering, Beihang University, Beijing, 100083 China; 3 Beijing Advanced Innovation Centre for Biomedical Engineering, Beihang University, Beijing, 100083 China

**Keywords:** Venous graft, Low-density lipoprotein, Hydraulic conductivity, Wall shear stress

## Abstract

**Background:**

Blood flow condition may have influence upon the hydraulic conductivity of venous graft (*L*_p,vein_) in an arterial bypass, then affecting the accumulation of low-density lipoproteins (LDLs) within the graft wall. To probe this possibility, we first measured in vitro the filtration rates of swine lateral saphenous vein segments under different flow rates, and the correlation of *L*_p,vein_ with wall shear stress (WSS) was then obtained.

**Results:**

The experimental results showed that when WSS was very low, *L*_p,vein_ would increase drastically with WSS from 1.16 ± 0.15 × 10^−11^ m/s Pa at 0 dyn/cm^2^ to 2.17 ± 0.20 × 10^−11^ m/s Pa at 0.7 dyn/cm^2^, then became constant of approximately 2.33 × 10^−11^ m/s Pa as the WSS increased further. Based on the experimental results, we assumed three different cases of *L*_p,vein_ and numerically simulated the LDLs transport in an arterial bypass model with venous graft. Case A: *L*_p,vein_ = 2.33 × 10^−11^ m/s Pa; Case B: *L*_p,vein_ = 1.16 × 10^−11^ m/s Pa (static condition with WSS of 0); Case C: *L*_p,vein_ was shear dependent. The simulation showed that the deposition/accumulation of LDLs within the venous graft wall in Case A was greatly enhanced when compared with that in Case B. However, the LDL accumulation in the graft wall was similar for Case A and Case C.

**Conclusions:**

Our study, therefore, indicates that when the venous graft was implanted as a bypass graft, the *L*_p,vein_ might remain nearly constant along its whole length except for very few areas where the value of WSS was extremely low (less than 0.7 dyn/cm^2^) and the effects of *L*_p,vein_ modulated by blood flow on LDL transport may be neglected.

## Introduction

Autogenous vein (e.g., the greater saphenous vein) segments are widely used in vascular bypass surgery to relieve arterial occlusion. Nevertheless, when implanted into the arterial system as grafts, veins will develop a rapidly progressive and structurally diffusive form of atherosclerotic lesions which has been termed as “accelerated atherosclerosis” [1–4], and this has become the major cause of venous graft late failure [5, 6].

The mechanism of the accelerated atherogenesis in venous grafts has been studied extensively [3, 7]. It is well documented that the process of atherogenesis in venous grafts is very similar to the one in the arterial system, in which the deposition/accumulation of lipids such as low-density lipoproteins (LDLs) within the arterial wall is the initial event, and the high LDL concentration within the vessel wall could accelerate the atherogenesis [8].

From the viewpoint of mass transport, the hydraulic conductivity of blood vessel walls may play an important role in the accumulation of LDLs and atherosclerotic lesion development in the venous graft. In our previous study [9], we measured hydraulic conductivity of the swine lateral saphenous vein in vitro. Using the measured data, we numerically analyzed the transport of LDLs in the venous graft. Our result demonstrated that hydraulic conductivity of the venous graft was significantly higher than that of the host artery. High hydraulic conductivity could not only elevate concentration polarization of LDLs at the luminal surface of the venous graft, and then enhancing the transport of LDLs into the venous graft wall by the mechanism of concentration gradient, but also could directly lead to high influx of LDLs into the vessel wall by convective flow. As a result, LDLs would accumulate rapidly within the wall of the venous graft, in turn accelerating the genesis and development of atherosclerosis in the venous grafts.

The hydraulic conductivity of arteries and cultured endothelial cells has shown to be greatly affected by fluid flow [10]. For instance, using bovine aortic endothelial cell (BAEC) monolayers, Sill et al. [11] observed that fluid flow could cause an increase in hydraulic conductivity when compared to that in the absence of flow. Lever et al. found approximately 30% increase in hydraulic conductivity of the rabbit common carotid artery when the flow rate was increased from zero to a level of 10 mL/min [12]. However, there is a paucity of investigations on the effect of flow conditions on the hydraulic conductivity of the vein.

Since flow conditions may also affect the hydraulic conductivities of the venous graft (*L*_p,vein_), in turn affecting LDL deposition/accumulation within the graft wall, in the present article, we measured in vitro the filtration rates of the swine lateral saphenous vein under different wall shear stress (WSS), from which the correlation of *L*_p,vein_ with wall shear stress was derived. We then numerically simulated LDL transport in a two-dimensional bypass model with a venous graft and analyzed the deposition and accumulation of LDLs within the wall of venous graft.

## Methods

### Measurement of filtration rates under different WSS for venous wall

#### Preparation of vessel segments

The lateral saphenous veins obtained from farm swine were used for the study. Experiments in the present study were approved by the university ethics review board. The specifically designed metal supporting frame was used to fix the 6- to 7-cm-long segment of blood excised from the swine leg. This procedure of the venous segment preparation for measurements has been described in detail previously [9].

#### Perfusion solution

For perfusion, freshly prepared albumin Krebs solution was used. Bovine serum albumin (Sigma Chemical Co., St. Louis, MO, USA) was dissolved in the Krebs solution (concentrations in mmol/L: NaCl, 118; KCl, 4.7; NaHCO_3_, 25; KH_2_PO_4_, 1.2; MgSO_4_, 1.2; CaCl_2_, 2.5; glucose, 11) at a concentration of 1.0 mg/mL (density, 1.005 g/cm^3^; viscous, 0.0116 g/cm s). The pH value of the solution was adjusted to 7.4.

#### Experimental procedure

**Fig. 1:**
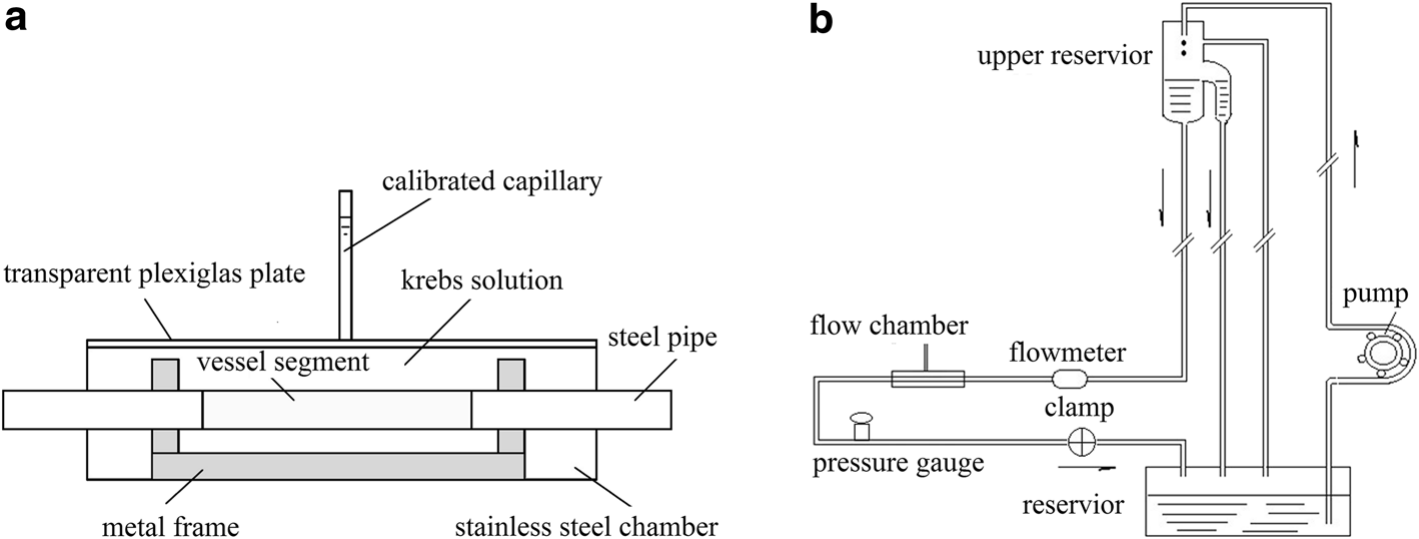
**a** Schematic drawing of the stainless steel chamber encasing the test blood vessel. **b** Schematic drawing of the experimental perfusion system

The harvested vein segment fixed by a metal frame was encased in the stainless steel chamber (Fig. 1a) and connected to the experimental perfusion system (Fig. 1b), in which the upper reservoir could provide a steady flow through the vessel keeping the vessel pressurized at a preset constant pressure. Then the stainless steel chamber was filled with the Krebs solution and covered by a transparent Plexiglas plate. A calibrated capillary was installed on the Plexiglas cover plate with its orifice connecting to the inside of the chamber for measuring the increment of liquid volume within the chamber.

The filtration rate measurements of the vein were carried out under arterial pressure (hydrostatic pressure 100 mmHg, transmural pressure 70 mmHg) [13] with different flow rates, i.e., 0 mL/min, 20 mL/min, 40 mL/min, 60 mL/min, 80 mL/min, 100 mL/min, 200 mL/min and 500 mL/min. By adjusting the clamp on the outlet tubing connected to the distal end of the test blood vessel, the desired flow rate through the vessel could be obtained.

The filtration rate (*v*_w_) across the test vessel wall was calculated by the following formula:

1 $$v_{\text{w}} = \Delta V/St,$$

where ∆*V* is the liquid volume between two marked lines on the calibrated capillary, *t* is the time required for the liquid meniscus rising in the capillary to pass the two marked lines and *S* is the total area of the outer surface of the test vessel.

Based on the *v*_w_, we could obtain the *L*_p,vein_ using Darcy’s law:

2 $$L_{\text{p,vein}} = V_{\text{w}} /\Delta p.$$

The wall shear stress (***τ***_w_) acting on the luminal surface of the test vessel was given by

3 $$\varvec{\tau}_{\text{w}} = 4\mu Q/\left( {\pi R^{ 3} } \right),$$

where *μ* and *R* represent, respectively, the viscosity of perfusion solution and the internal radius of the test vessel. *Q* is the flow rate through the test vessel.

Each experiment with a given flow rate was repeated three times and all experiments were carried out at a room temperature of 23 ± 0.5 °C. During the perfusing process, the length and external diameter of the vessel were measured for calculating the total area *S* of the vessel. With finishing each experiment turn, the test vessel was fixed at the test hemodynamics condition by perfusing with 10% formalin solution overnight to obtain the histological cross sections of the vessel, from which the internal radius *R* of the test vessel could be established.

#### Statistical analysis

SPSS19.0 (SPSS Inc.) software was applied for statistical analysis. All experiment results were expressed as mean ± standard deviation (SD). One-way ANOVA was used to compare mean values of the experiment data. Differences were considered significant when *P *< 0.05.

### Numerical simulation of LDL transport

#### Geometric model

**Fig. 2:**
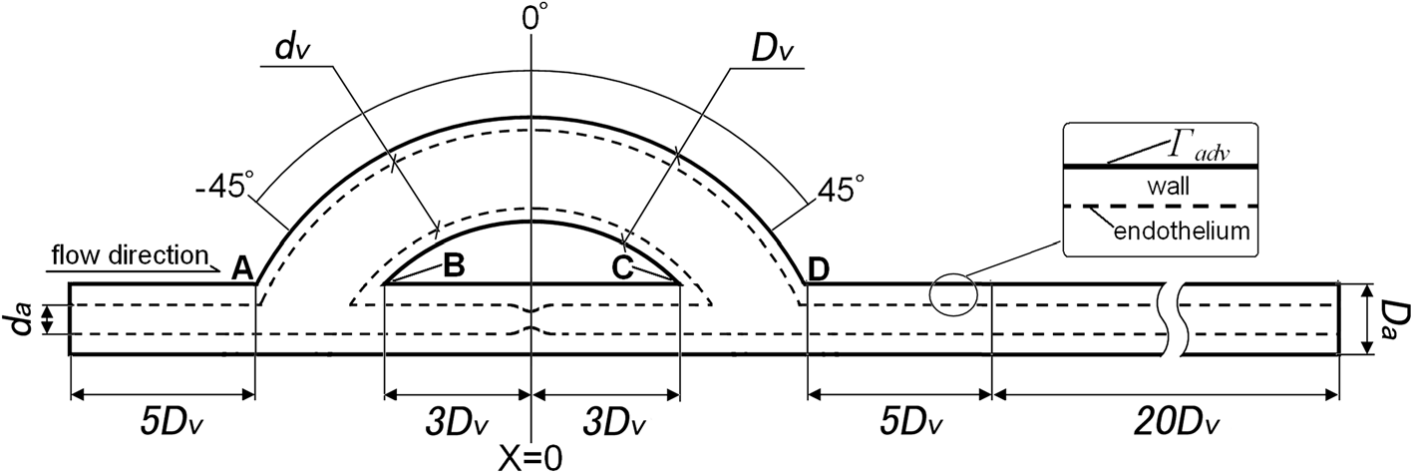
Schematic illustration of the bypass model with venous graft used in the simulations

Simplified bypass models (two dimensional) with 80% area occluded in the host artery were adopted in this simulation, as shown in Fig. 2. In the calculated model, both the host artery wall and the venous graft wall were treated as a single-layer porous medium with an endothelium layer. $\varGamma_{\text{adv}}$ was the interface between the media and adventitia. The outer diameter of the host artery (*D*_a_) and the thickness of arterial wall (*h*_a_) were set to be 5.2 and 0.35 mm, respectively. The venous graft’s outer diameter and thickness (*D*_v_ and *h*_v_) were, respectively, 6.2 and 0.035 mm. The anastomosis angle was 45°. The host artery was 80% area occluded at the middle between points B and C.

#### Governing equations

To model bulk blood flow in the lumen and transmural flow in the endothelium layer, Navier–Stokes equations and Darcy’s law were employed, respectively. For modeling of mass balance, the convection–diffusion equation was employed in the lumen. To form a convection–diffusion–reaction equation in the endothelium layer, additional reaction term was added. Furthermore, the Kedem–Katchalsky equations were employed to couple the fluid dynamics and mass balance at membranes, i.e., endothelium.

##### Fluid flow dynamics

Blood flow in the vessel lumen was simulated by the steady-state incompressible Navier–Stokes and continuity equations [14]:

4 $$\rho (\varvec{u} \cdot \nabla )\varvec{u} + \nabla p - \mu \Delta \varvec{u} = \textbf{0,}$$

5 $$\nabla \cdot \varvec{u} = \textbf{0,}$$

where ***u*** and *p*, respectively, represent fluid velocity vector and pressure. *ρ* and *μ* are the density (*ρ *=1050 kg/m^3^) and the viscosity of blood (*μ *= 3.5 × 10^−3^ kg/m s), respectively [15]

The transmural fluid flow across the endothelium (*J*_v_) was modeled by the Kedem–Katchalsky equation [16]:

6 $$J_{\text{v}} = L_{{\text{pend}}} (\Delta p - \sigma \Delta \pi ),$$

where $\Delta p$ and $\Delta \pi$ are the hydraulic pressure and osmotic pressure differences across the endothelium; *σ* and *L*_pend_, respectively, represent the osmotic reflection coefficient and the hydraulic conductivity of the endothelium. In the present simulation, *∆π* was neglected because the influence of osmotic pressure on the fluid dynamics is too small, compared to hydraulic pressure.

The transmural fluid flow across the arterial wall and venous wall was modeled by Darcy’s law.

In a homogeneously permeable medium, Darcy’s law can be described as a simple proportional relationship between the instantaneous flow rate through a porous medium of permeability, the dynamic viscosity of the fluid and the pressure drop over a given distance. From the viewpoint of mass transport, Darcy’s law can be used to describe the hydraulic conductivity of blood vessel walls when the blood flow remains stable, laminar:

7 $$\frac{{\mu_{\text{w}} }}{{{\rm K}_{\text{w}} }}\varvec{u}_{\text{w}} { = } - \nabla (p_{\text{w}} )$$

and continuity equation

8 $$\nabla \cdot \varvec{u}_{\text{w}} = \textbf{0},$$

where ***u***_w_ and ***p***_w_ represent, respectively, the velocity vector and pressure in the vessel wall, *K*_w_ is the hydraulic permeability of the wall and *μ*_w_ is the viscosity of plasma.

##### Mass transport

The transport of LDLs in the vessel lumen was modeled by the convection–diffusion equation as follows [17]:

9 $$\nabla \cdot ( - D\nabla c + c\varvec{u}) = \textbf{0},$$

where *c* represents the concentration of LDLs in the lumen, and *D* is the diffusion coefficient of LDLs in blood, *D *= 5.898 × 10^−12^ m^2^/s [18].

The flux of LDLs across the endothelium (*J*_s_) was described by the following equation [17]:

10 $$J_{\text{s}} = P_{{\text{end}}}\Delta c + (\text{1} - \sigma_{\text{f}} )J_{\text{v}} \bar{c},$$

where ∆*c* is LDL concentration difference across the endothelium, *P*_end_ is the endothelial permeability to LDLs and $\bar{c}$ is the mean concentration of LDLs in the endothelium. *σ*_f_ is the solvent reflection coefficient.

The transport of LDLs within the vessel wall was modeled by the convection–diffusion–reaction equation as follows [19]:

11 $$\nabla \cdot ( - D_{\text{w}} \nabla c_{\text{w}} + K_{{\text{lag}}} c_{\text{w}} \varvec{u}_{\text{w}} ) = r_{\text{w}} c_{\text{w}} ,$$

where *c*_w_ and *D*_w_, respectively, represent the concentration of LDLs within vessel wall and the effective diffusivity of LDLs in the wall, *r*_w_ is the chemical reaction rate, and *K*_lag_ is the LDLs lag coefficient.

#### Boundary conditions

##### Boundary conditions for blood flow simulation

A fully developed (parabolic) velocity profile with a mean velocity of 0.22 m/s was applied at the inlet of the arterial lumen so that Reynolds number (*Re*) based on the internal diameter of the host artery was 300. At the outlet of arterial lumen, the pressure *p* was set at 100 mmHg. At the lumen side of the endothelial boundary, a wall to lumen transmural fluid velocity was prescribed by

12 $$\varvec{u} \cdot \varvec{n}_{\text{l}} = J_{\text{v}} .$$

At the wall side of the endothelial boundary, a lumen-to-wall transmural velocity was prescribed by

13 $$\varvec{u}_{\text{w}} \cdot \varvec{n}_{\text{w}} = - J_{\text{v}} ,$$

where ***n***_l_ and ***n***_w_ represent the out normal vectors of the lumen sub-domain and wall sub-domain, respectively. *J*_v_ is the transmural fluid velocity across the endothelium.

A constant pressure boundary condition of 30 mmHg was prescribed at the media–adventitia interface (*Γ*_adv_) [19].

At the occlusion of the host arterial lumen:

14 $$\varvec{u} = 0.$$

No flow was assumed for all other boundaries.

##### Boundary conditions for LDL transport

The inflow concentration of LDLs (*c*_0_) at the lumen inlet boundary was taken as 28.6 × 10^−3^nmol/mm^3^ [20]. At the occlusion of the arterial lumen, the flux of LDLs in the normal direction of the lumen domain was set at 0. At the lumen side of the endothelial boundary, a wall to lumen flux of LDLs was prescribed by

15 $$- D\nabla c\varvec{n}_{\text{l}} + \varvec{u}c\varvec{n}_{\text{l}} = J_{\text{s}} .$$

At the wall side of the endothelial boundary, a lumen-to-wall flux of LDLs was prescribed by

16 $$- D_{\text{w}} \nabla c_{\text{w}} \varvec{n}_{\text{w}} + \varvec{u}_{\text{w}} c_{\text{w}} \varvec{n}_{\text{w}} = - J_{\text{s}} .$$

For other boundaries, the concentration gradient of LDLs in the boundary normal direction was assumed to be zero.

#### Parameters

To solve the governing equations, we need to acquire the values of seven parameters, namely the hydraulic permeability of subendothelial wall (*K*_w_), the hydraulic conductivity of endothelium (*L*_pend_), the solvent reflection coefficient (*σ*_f_), the endothelial permeability (*P*_end_) to LDLs, the chemical reaction rate (*r*_w_), the effective diffusivity of LDLs in the subendothelial layer (*D*_w_) and the solute lag coefficient (*K*_lag_).

##### Host artery

All the parameters were adopted in the literature, which are presented in Table 1.

**Table 1:** Values of parameters used in the simulation

Symbol	Description	Value, units
*D*_v_	Outer diameter of venous graft [9]	6.2 mm
*d*_v_	Internal diameter of venous graft [9]	6.13 mm
*h*_v_	Thickness of venous wall [9]	0.035 mm
*D*_a_	Outer diameter of host artery [9]	5.2 mm
*d*_a_	Internal diameter of host artery [9]	4.5 mm
*h*_a_	Thickness of arterial wall [9]	0.35 mm
*Ρ*	Density of blood [15]	1050 kg/m^3^
*µ*	Viscosity of blood [15]	3.5 × 10^−3^ kg/m s
*L*_pend,artery_	Hydraulic conductivity of arterial endothelium	3.5551 × 10^−12^ m/s Pa
*L*_pend,vein_	Hydraulic conductivity of venous endothelium	
	*Case A:* 3.9091 × 10^−11^ m/s Pa	
	*Case B:* 1.4457 × 10^−11^ m/s Pa	
	*Case C:* (2.522 × 10^−11^) × WSS^(1/10)^ + 1.532 × 10^−11^	
*µ*_w, artery_	Viscosity of plasma in arterial wall [23]	0.72 × 10^−3^ kg/m s
*µ*_w, vein_	Viscosity of plasma in venous wall [23]	0.72 × 10^−3^ kg/m s
*K*_w, artery_	Hydraulic permeability of subendothelial wall of artery	1.3438 × 10^−18^ m^2^
*K*_w, vein_	Hydraulic permeability of subendothelial wall of vein	1.4572 × 10^−18^ m^2^
*D*_l_	LDL diffusion coefficient in luminal blood [18]	5.898 × 10^−12^ m^2^/s
*D*_w, artery_	LDL diffusion coefficient in arterial wall [17, 24]	1.42 × 10^−12^ m^2^/s
*D*_w, vein_	LDL diffusion coefficient in venous wall [17, 24]	1.42 × 10^−12^ m^2^/s
*P*_end, artery_	Arterial endothelial permeability [17, 24]	5.21 × 10^−10^ m/s
*P*_end, vein_	Venous endothelial permeability [17, 24]	5.21 × 10^−10^ m/s
*σ*_f, artery_	Solvent reflection coefficient in arterial wall [23, 25]	0.997
*σ*_f, vein_	Solvent reflection coefficient in venous wall [23, 25]	0.997
*K*_lag, artery_	Solute lag coefficient in arterial wall [17, 24]	0.1486
*K*_lag, vein_	Solute lag coefficient in venous wall [17, 24]	0.1486
*r*_w, artery_	LDL chemical reaction rate in arterial wall [17, 24]	− 6.05 × 10^−4^ s^−1^
*r*_w, vein_	LDL chemical reaction rate in venous wall [17, 24]	− 6.05 × 10^−4^ s^−1^

##### Venous graft

The hydraulic conductivity of the venous wall (*L*_p,vein_), hydraulic conductivity of the venous endothelium (*L*_pend,vein_) and hydraulic conductivity of the subendothelial wall of the vein (*L*_pw,vein_) were assumed to have the relation below [21]:

17 $$\frac{\text{1}}{{L_{{\text{p,vein}}} }} = \frac{\text{1}}{{L_{{\text{pend,vein}}} }} + \frac{\text{1}}{{L_{{\text{pw,vein}}} }},$$

where *L*_p,vein_=* V*_w_/∆*p*, which could be obtained from the results of “Measurement of filtration rate for venous wall”. We assumed that the *L*_pw,vein_ was 4 folder of *L*_pend, vein_ when we performed the test experiments on vessels under no flow condition [19], then *L*_pw,vein_ and *L*_pend,vein_ under 0 dyn/cm^2^ of WSS could be acquired. Assuming that the *L*_pw,vein_ is constant with the flow rate, we could obtain *L*_pend,vein_ under other different WSS conditions.

Since $L_{{\text{pw,vein}}} = \frac{{K_{{\text{w,vein}}} }}{{\mu_{{\text{w,vein}}} h_{{\text{vein}}} }}$ [22], and *h*_vein_ (the thickness of venous wall) could be obtained from the histological cross sections, we can get the hydraulic permeability of subendothelial wall of vein (*K*_w,vein_). We assumed that other parameters of the vein were the same as those of the artery (Table 1).

#### Computation procedures

The numerical simulations were carried out using a validated finite elemental algorithm Comsol Multiphysics (COMSOL AB, Sweden). First, the pressure and the velocity fields were obtained by performing flow simulations, the solutions were used later on for the simulations of LDL transport. The threshold value for velocity and continuity residual detection was set to 1.0 × 10^−5^ to ensure the convergence of the calculated results. The numerical results for the flow and the transport of LDLs were determined to be mesh independent. In addition, we validated the mesh independence of results obtained. The criteria of mesh independence were set as the difference of *J*_s_ between the meshes used for computations and denser meshes was less than 3%. Based on these criteria, the final computational meshes consisted of 712,620 cubic elements.

## Results

### Hydraulic conductivity

**Fig. 3:**
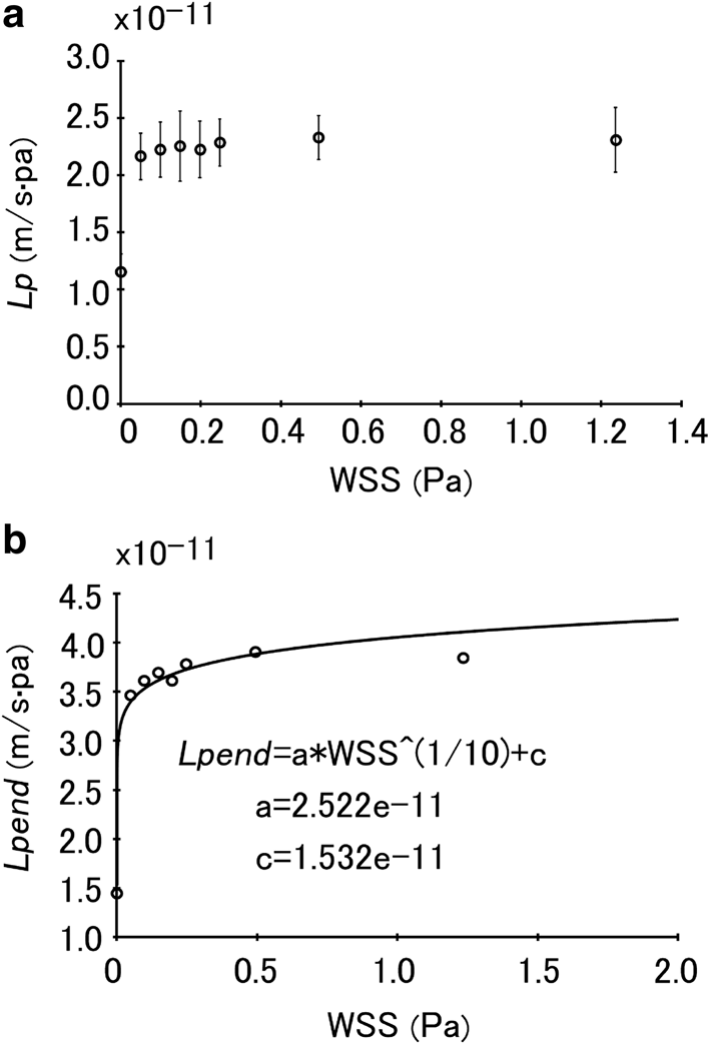
**a** Hydraulic conductivities (*L*_p,vein_) of the venous segments measured under different wall shear stress conditions. **b** Hydraulic conductivities of the endothelium of the venous segments (*L*_pend,vein_) under different wall shear stress conditions

In the present study, we first measured the filtration rates across (*V*_w_) the walls of the venous segments under different WSS conditions. Then based on the measured data, we derived the correlation of *L*_p,vein_ with WSS using Eq. (2). The results are shown in Fig. 3a. It can be seen that when WSS is very low *L*_p,vein_ would increase dramatically from 1.16 ± 0.15 × 10^−11^ m/s Pa at WSS of 0 to 2.17 ± 0.20 × 10^−11^ m/s Pa at WSS of 0.7 dyn/cm^2^. But beyond that *L*_p,vein_ would remain almost constant (approximately 2.33 × 10^−11^ m/s Pa).

Based on the values of *L*_p,vein_, we calculated *L*_pend,vein_, the hydraulic conductivity of the venous endothelium using Eq. (17). The obtained results are shown in Fig. 3b. By curve fitting, it can be shown that *L*_pend,vein_= 2.522 × 10^−11^ × WSS^0.1^ + 1.532 × 10^−11^.

### Numerical comparison of LDL transport

Since the experimental measurements showed that when WSS was greater than 0.7 dyn/cm^2^, *L*_p,vein_ would remain almost constant at a value of approximately 2.33 × 10^−11^ m/s Pa and the numerical simulation of blood flow indicated that there were very few areas in the bypass model where WSS would be less than 0.7 dyn/cm^2^, in the present numerical study, we assumed three cases of *L*_p,vein_ to compute the transport and accumulation of LDLs in the bypass mode. Case A: *L*_p,vein_ = a constant value of 2.33 × 10^−11^ m/s Pa; Case B: *L*_p,vein_ = 1.16 × 10^−11^ m/s Pa (static condition with WSS of 0); Case C: *L*_p,vein_ is shear dependent as derived from the experimental measurements.

**Fig. 4:**
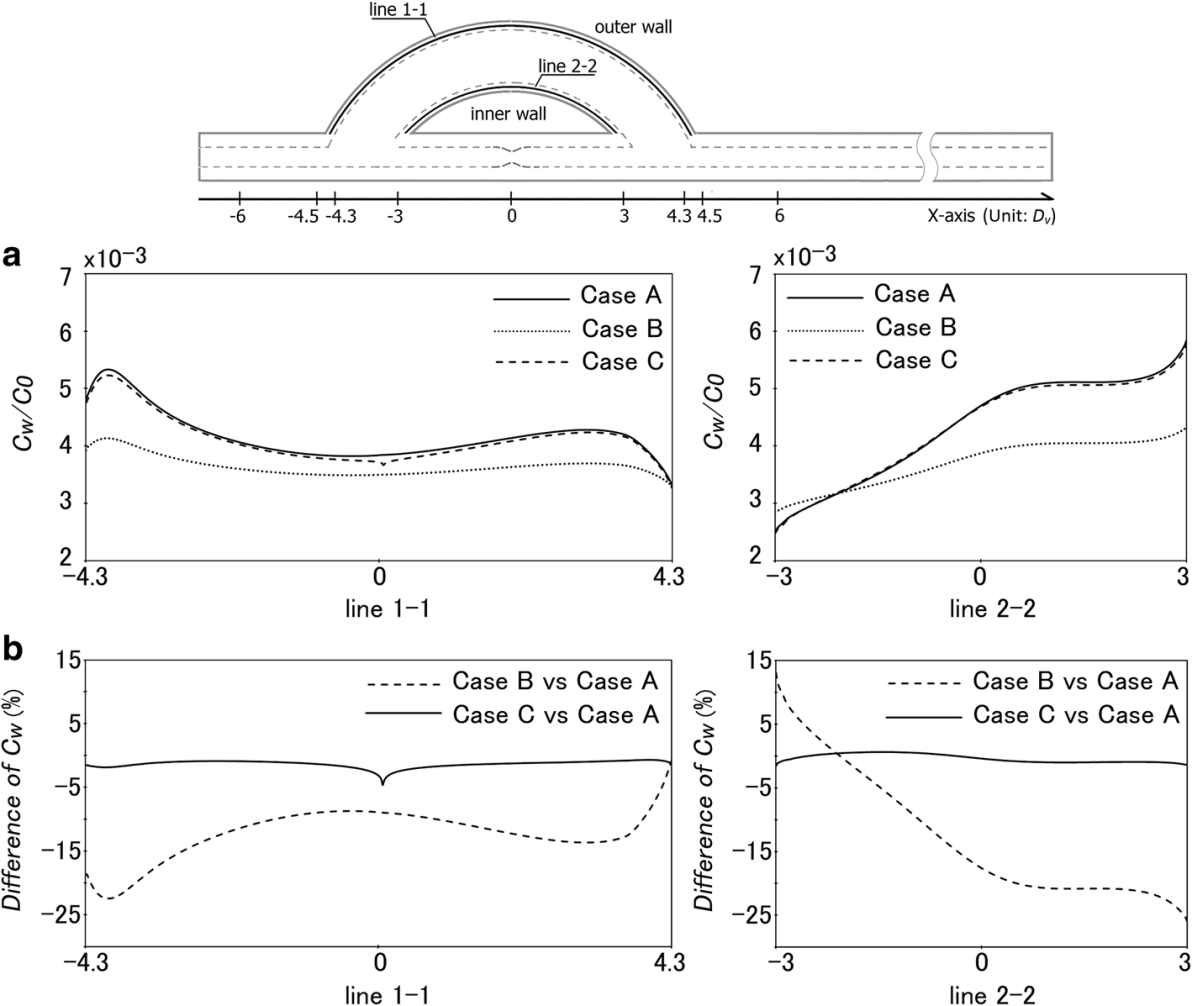
Numerical comparison of *c*_w_ (normalized by *c*_0_) along the inner and outer walls of the venous graft for Cases A, B and C. **a** Distribution of *c*_w_ along the middle wall of the venous graft. **b** Percentage difference of *c*_w_ in the venous graft among the three cases, which is defined as (*c*_w,Case B_ − *c*_w,Case A_)/*c*_w,Case A_ or (*c*_w,Case C_ − *c*_w,Case A_)/*c*_w,Case A_

Figure 4a shows LDL concentration (*c*_w_) distributions in the middle position of the venous graft wall for the three cases. As evident from the figure, the distribution of *c*_w_ for Case A and Case C is very similar. However, when compared with Case B, *c*_w_ for Cases A and C is generally higher in most regions of the venous graft wall. Figure 4b gives the comparison of *c*_w_ among the three cases in terms of percentage difference. It can be seen that the difference between Case A and Case B is prominent. For instance, the percentage difference is more than 10% in most regions, especially in the exit region of the graft inner wall (line 2-2), where the percentage difference reaches as high as 26%. But for Case A and Case C, there is no evident difference in all regions of the graft. The highest difference in *c*_w_ between the two cases is located in the middle section of the outer wall (line 1-1), which is less than 5%.

### Effect of arterial stenosis severity on LDL transport

In clinical practice, the stenosis degree is an important indicator of necessity for bypass surgery. Usually, when the degree of arterial narrowing is greater than 70%, surgical intervention would be eminent. Besides, after the surgery, the stenosis of the host artery would continue to develop, resulting in the alteration of the flow field in the venous graft, which would, in turn, affect the transport of LDLs in the graft. To clarify the impact of stenosis severity on LDL deposition/accumulation within the venous graft wall, we numerically simulated LDL transport in bypass models with different arterial stenosis that has 70, 80 and 90% reduction in the cross-sectional area of the host artery, using the shear-dependent *L*_p,vein_.

**Fig. 5:**
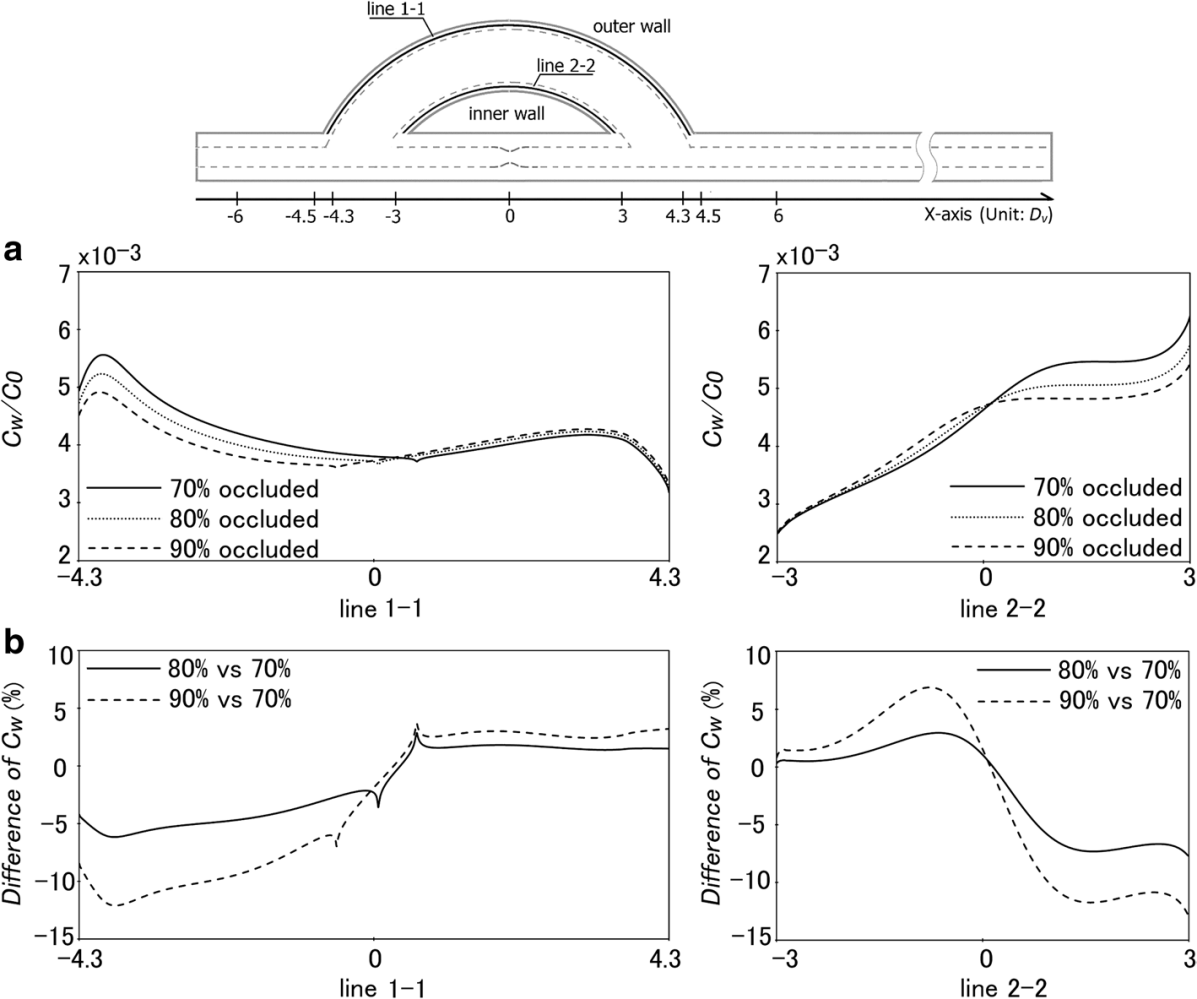
Numerical results of *c*_w_ (normalized by *c*_0_) along different lines for cases with stenosis degrees of 70%, 80% and 90%. **a** Distribution of *c*_w_ in the venous graft. **b** Percentage difference of *c*_w_ in the venous graft. The percentage difference was defined as (*c*_w, 80% occluded_ − *c*_w, 70% occluded_)/*c*_w, 70% occluded_ or (*c*_w, 90% occluded_ − *c*_w, 70% occluded_)/*c*_w, 70% occluded_

As shown in Fig. 5a, the distributions of *c*_w_ along the venous graft have much in common for the three bypass models with different stenoses. Nevertheless, it is still evident that the positions of the local minima of *c*_w_, locating at the middle of the graft outer wall, are different. The percentage differences in the *c*_w_ for the three models are presented in Fig. 5b. From the figure, it can be seen that there is no significant difference in *c*_w_ between the models of 70 and 80% occlusion. Only when comparing the 90% model with the 70% one, the percentage difference is somewhat prominent, the maximum of which is approximately 15%.

## Discussion

The late failure of venous grafts, which is usually caused by accelerated atherogenesis, has long been a common problem that limits the therapy of arterial bypass surgery. We previously demonstrated that enhanced *L*_p,vein_ could lead to fast infiltration/deposition of LDLs within the venous graft wall, and hence the accelerated genesis and development of atherosclerosis [9].

As hydraulic conductivity of blood vessels can be affected by blood flow, in the present study we investigated the effect of *L*_p,vein_ modulated by flow on LDL accumulations within the wall of the venous graft. We first measured the filtration rates of the swine lateral saphenous vein (hence the *L*_p,vein_) under different flow/WSS conditions. To our best knowledge, this is the first in vitro study of its kind to clarify the shear dependence of venous wall hydraulic conductivity, though a few studies have investigated the hydraulic conductivities of endothelial cell monolayers, capillaries and arterial vessels [10, 12, 26–29]. From our experimental results, it was found that the luminal flow in the venous vessels investigated could cause a significant increase in *L*_p,vein_ when compared with the case of flow absence. This finding was consistent with the studies performed on arteries, capillaries and endothelial cell monolayers by others, which indicated that flow shear could induce increases in hydraulic conductivity via a NO-dependent mechanism [10, 30]. But, our results showed that the increase in hydraulic conductivity of venous segments induced by a change of WSS (from zero to a steady level of more than 0.7 dyn/cm^2^) was approximately 100%, which was much higher than the results obtained on intact arterial vessels (e.g., the excised rabbit carotid artery [12] that exhibited only 30% increase in hydraulic conductivity when WSS elevated from 0 to 1 dyn/cm^2^), but was lower than those obtained on endothelial cell monolayers (e.g., exposure of cultured bovine aortic endothelial monolayers to a WSS of 12 dyn/cm^2^ elicited a 2.37-fold increase in hydraulic conductivity [10]). This discrepancy might be attributed to the structural differences in the subendothelial layers of the test models. It had been documented that the subendothelial layers of blood vessels, including the intima and media, contribute most of the overall resistance to filtration flow across the vessel wall [31, 32]. Considering that the endothelial cell monolayers have no subendothelial layer and the subendothelial layer in the artery is thicker and compacter than that of the vein, the sensitivity of the venous wall hydraulic conductivity in response to WSS should be between those of the arterial wall and the endothelial cell monolayers.

Based on the obtained hydraulic conductivity, we simulated numerically the transport of LDLs within the venous graft of an arterial bypass model. It had been documented that LDL transport, including the transport in the lumen (from the bulk blood flow to the endothelium) and the trans-endothelial transport (across the endothelium), was mainly affected by hydraulic conductivity [18]. Indeed, our numerical results revealed a significant difference in *c*_w_ between Case A (with flow and *L*_p,vein_ = 2.33 × 10^−11^ m/s Pa) and Case B (with no flow of *L*_p,vein_= 1.16 × 10^−11^ m/s Pa). This finding suggests that flow could affect *L*_p,vein_, then affecting LDL deposition/accumulation within the graft wall.

In addition, our result showed that there was no evident difference in *c*_w_ between Case A and Case C (in which *L*_p,vein_ was assumed to be shear dependent), indicating that the influence of shear-dependent *L*_p,vein_ on LDL transport was marginal. This seems different from some previous studies that demonstrated a significant influence of hydraulic conductivity with shear-dependent nature on LDL transport. For instance, using an idealized model of a stenosed artery, Sun et al. [19] found that when compared with the independent case, the shear-dependent *L*_p_ could lead to apparent differences in LDL concentration within the subendothelium, in which the difference could reach as high as 12%. We suppose that the discrepancy between our results and others could be explained as follows. Different from the artery, hydraulic conductivity of the vein was almost independent of the flow-induced wall shear stress except for when the WSS was extremely low. Since there were very few regions in the bypass model with very low WSS, the hydraulic conductivities of the venous graft wall for Case C (the shear-dependent case) had to be similar to that in Case A (the independent one).

In the present study, we also investigated the effect of stenosis severity of the host artery on LDL infiltration/accumulation within the venous graft wall. The results showed that there were some moderate differences in *c*_w_ among the cases with different stenosis in the host artery. When compared the 90% occlusion model with the 70% one, the maximum percentage difference in the *c*_w_ reaches 15%. We believe that the effect of the stenosis severity on *c*_w_ is mostly caused by the luminal blood flow per se rather than the filtration rate across the graft wall because the hydraulic conductivity of the venous graft is almost constant along the venous graft.

Here, it should be mentioned that to simplify the simulation, the complex heterogeneous structure of the vessel wall was approximated by a simple homogeneous layer. In recent years, multilayer models were used to characterize LDL transport in blood vessel walls [18, 33, 34], which could better describe the distribution of LDLs in the venous graft. One thing that should be addressed is that the hemodynamic condition can be influenced by several factors (individual geometric parameters, material parameters, realistic blood flow condition). Investigators have paid much more attention to these aspects. For example, Peng et al. investigated the correlation between hemodynamic parameters and individual geometric factors in the patient-specific coronary arteries, and their results showed that the increasing severity of the stenosis was associated with the increased maximum wall shear stress at the stenosis region [35]. Xiong et al. calculated the blood flow field distribution in idealized 2D models, and results showed that high pressure drop would create a pathological WSS environment for the further growth of the plaque [36]. Liu et al. investigated the hemodynamic alteration in the cerebral circulation and revealed that the structural status could provide comprehensive information about the hemodynamic alterations in the pathological circle of Wills arterial structures [37]. Zhang et al. introduced a pressure-based carotid arterial functional assessment index generated from computational fluid dynamic simulation based on digital subtracted angiography (DSA) data. Their results showed that the realistic blood condition obtained through invasive approach would remain one efficient way to study the relationship between hemodynamic disorder caused by internal carotid artery (ICA) stenosis and subsequent perfusion variations in brain [14]. Based on their investigations, it can be addressed that several significant parameters in terms of realistic geometry, artery material and flow condition would certainly influence the hemodynamic behavior, and then influence the LDL transport within the blood artery from the viewpoint of mass transport. Though this can be significant limitations for our study, our study remains a preliminary study as we focus on the correlation between the wall shear stress and hydraulic conductivity of venous endothelium, and its influence on the transport phenomenon of LDL. Further studies based on more comprehensive models and realistic blood conditions still need to be carried out to strengthen our study.

## Conclusion

To probe the possibility that the blood flow condition would affect the accumulation of LDLs within the graft by influencing the hydraulic conductivity of venous graft in arterial bypass, LDL transport phenomenon in a two-dimensional bypass model with a venous graft was numerically simulated, while the deposition and accumulation of LDLs within the wall of venous graft were analyzed based on the filtration rates of the swine lateral saphenous vein under different WSS measured from the in vitro experiments. Our results indicate that the blood flow would affect the transport and accumulation of LDLs within the venous graft wall when the WSS is extremely low, as the *L*_p,vein_ can be significantly influenced by the WSS with the range of 0–0.7 dyn/cm^2^. While when WSS is above 0.7 dyn/cm^2^, the influence of shear-dependent *L*_p,vein_ on LDL transport is marginal. Since the low WSS (< 0.7 dyn/cm^2^) areas remain few, it can be addressed that the effect of *L*_p,vein_ modulated by blood flow on LDL transport remains weak.

## Abbreviations

LDLs: low-density lipoproteins
WSS: wall shear stress
BAEC: bovine aortic endothelial cell
SD: standard deviation
*Re*: Reynolds number
DSA: digital subtracted angiography
ICA: internal carotid artery
